# The moderating effect of cognitive reserve on cognitive function in patients with Acute Ischemic Stroke

**DOI:** 10.3389/fnagi.2022.1011510

**Published:** 2022-11-16

**Authors:** Fanfan Li, Xiangjing Kong, Huanzhi Zhu, Hanzhang Xu, Bei Wu, Yanpei Cao, Juan Li

**Affiliations:** ^1^School of Nursing, Naval Medical University, Shanghai, China; ^2^Air Force Hospital of Eastern Theater Command, Nanjing, China; ^3^School of Nursing, Duke University, Durham, NC, United States; ^4^School of Medicine, Duke University, Durham, NC, United States; ^5^Rory Meyers College of Nursing, New York University, New York, NY, United States; ^6^Department of Nursing, Huashan Hospital, Fudan University, Shanghai, China; ^7^National Center for Neurological Disorders, Shanghai, China

**Keywords:** Acute Ischemic Stroke, cognitive reserve, moderate effect, cognitive function, cognitive impairment, Cognitive Reserve Index questionnaire

## Abstract

**Background:**

Recovery of cognitive function after stroke has inter-individual variability. The theory of cognitive reserve offers a potential explanation of the variability in cognitive function after stroke.

**Objective:**

This study aimed to investigate the moderating effect of cognitive reserve on the relationship between the stroke severity and cognitive function after stroke.

**Materials and methods:**

A total of 220 patients with Acute Ischemic Stroke (AIS) were recruited in 2021 from two stroke centers in Nanjing, China. The National Institutes of Health Stroke Scale (NIHSS) was used to assess stroke severity upon admission. Cognitive Reserve Index questionnaire (CRIq) and validated Montreal Cognitive Assessment, Changsha Version (MoCA-CS) were used to assess cognitive reserve and cognitive function within 7 days after stroke onset, respectively. A series of multivariate linear regression models were applied to test the moderating effect of cognitive reserve.

**Results:**

Patients with a higher level of cognitive reserve had better cognitive function after stroke compared with those with a lower level of cognitive reserve (β = 0.074, *p* = 0.003). The interaction of NIHSS and cognitive reserve was statistically significant (β = −0.010, *p* = 0.045) after adjusting for some key covariates [e.g., age, marital status, Oxfordshire Community Stroke Project (OCSP) classification, Trial of ORG 10172 in Acute Stroke Treatment (TOAST) classification, cerebral vascular stenosis, diabetes and atrial fibrillation].

**Conclusion:**

Cognitive reserve may help to buffer the effect of stroke-related pathology on cognitive decline in Chinese acute stroke patients. Enhancing cognitive reserve in stroke patients may be one of the potential strategies for preventing vascular dementia.

## Introduction

Stroke is the second leading cause of death in the world (Stark et al., 2021). The incidence of stroke in China was 246.8 per 100,000 person-years and death rate for cerebrovascular diseases was 149.49 per 100,000 (Wang et al., 2020). Therefore, stroke is characterized as having high morbidity, high mortality, and high recurrence rate, and has significant economic burden (Wang et al., 2020). Acute Ischemic Stroke (AIS) is the most common type of stroke, accounting for 69.6–70.8% of all stroke cases in China (Wang D. et al., 2017; Wang W. et al., 2017). About 53–81% of stroke survivors in China suffer from post-stroke cognitive impairment (PSCI) (Qu et al., 2015; Ding et al., 2019), which is characterized as experiencing impairment in at least one cognitive domains including executive function, attention, memory, language, and/or visuospatial function (Mok et al., 2017). PSCI affects the quality of life and survival time of stroke patients, and increases the subsequent incidence of dementia (Claesson et al., 2005; Melkas et al., 2009; Savva and Stephan, 2010). Therefore, it is critical to identify strategies to prevent PSCI in patients with AIS.

Cognitive reserve refers to the ability of the brain to maintain optimal cognitive functions by mobilizing pre-existing neural networks or reconstructing alternative neural networks to resist pathological damage (Stern, 2002; Barulli and Stern, 2013). A few studies have showed that cognitive reserve may explain the mismatch between the degree of pathological brain damage and clinical outcomes among some patients (e.g., cognition/motor function) (Katzman et al., 1988; Stern, 2002). Specifically, cognitive reserve shapes the brain’s capacity to compensate for pathological damage through neural compensation (e.g., recruiting uninjured brain functional areas), which moderates the impact of pathological damage on clinical manifestations (Stern, 2002), and varies among different individuals (Duda et al., 2014; Stenberg et al., 2020).

Prior studies on cognitive reserve mostly focused on Alzheimer’s disease and mild cognitive impairment among older adults (Yaffe et al., 2011; Liu et al., 2013; Ko et al., 2022). Findings from previous studies suggest that higher cognitive reserve can tolerate a more severe pathological burden or age-related changes and maintain a better cognitive function when there are pathophysiological changes in the brain. Emerging research has suggested a simplified methodological model of cognitive reserve in stroke patients, and claims that the cognitive reserve theory is also suitable for patients with stroke (Steffener et al., 2011; Steffener and Stern, 2012; Rosenich et al., 2020). In brief, this model includes three components: cognitive reserve, stroke-related pathological burden, and clinical outcomes, and the model demonstrates that cognitive reserve moderates the relationship between pathology of stroke and clinical outcomes after adjusting for confounding factors (e.g., age, sex, and SES) (Cizginer et al., 2017).

Several studies have assessed cognitive reserve in stroke patients and found lower levels of cognitive reserve was associated with more severe PSCI and a slower rate of post-stroke recovery (Ojala-Oksala et al., 2012; Shin et al., 2020). However, these two studies only used static and gross proxies (i.e., years of education and/or occupation) to measure cognitive reserve, which could not reflect the dynamic feature of cognitive reserve. Prior studies define cognitive reserve as a dynamic process over the lifespan that is influenced by both early life static factors (e.g., intelligence, level or years of education, occupational attainment, and socioeconomic status) as well as dynamic lifestyle activities (e.g., social activities, physical activity, and recreational activities) (Barulli and Stern, 2013). Therefore, cognitive reserve measured by more comprehensive instruments, such as the Cognitive Reserve Index questionnaire (CRIq), that capture both the static and dynamic aspects of cognitive reserve, can provide a comprehensive assessment of cognitive reserve (Gil-Pagés et al., 2019). To date, only one study used the CRIq to measure cognitive reserve in convalescent stroke patients and found that patients with higher levels of cognitive reserve had a lower prevalence of cognitive impairment after stroke (Abdullah et al., 2021). However, this study did not investigate whether the impact of cognitive reserve on cognitive function is the same across different stroke severity.

To address this knowledge gap, we aimed to investigate whether cognitive reserve moderates the relationship between stroke severity and cognitive function in Chinese stroke patients at an acute stage. We hypothesize that higher cognitive reserve in the stroke patients in a acute stroke phase is associated with better cognitive function after stroke, and cognitive reserve moderates the impact of stroke severity on cognitive function.

## Materials and methods

### Participants

This cross-sectional study was conducted by using convenience sampling. We recruited patients with AIS between July and November 2021 from two large stroke centers in Nanjing, China. Patients were eligible if they were: (1) diagnosed with AIS by a neurologist or a neurosurgeon based on a focal neurologic deficit and a corresponding infarct on magnetic resonance imaging (MRI); (2) aged ≥ 18 years at stroke onset; and (3) admitted to a stroke center within 7 days after onset. Exclusion criteria were: (1) having a transient ischemic attack (TIA); (2) having mental health diseases or dementia prior to stroke onset; and (3) having dysphasia or severe visual or hearing impairment. A total of 220 eligible patients with AIS were recruited in this study.

### Procedures

Two trained research assistants recruited patients for this study. Two trained neurologists assessed patients’ stroke severity and cognitive function. The research assistants interviewed patients or their primary family caregivers (Nucci et al., 2012)to assess cognitive reserve of patients. All patients and their primary family caregivers provided written informed consent. Clinical characteristics of patients were obtained from the medical records.

### Materials

#### Cognitive Reserve Index questionnaire

Cognitive Reserve Index questionnaire was used to assess cognitive reserve in AIS patients (Nucci et al., 2012). It includes three domains: (1) education, measured by years of formal and informal education; (2) occupation, classified into five levels depending on the cognitive load required for the job and the number of years spent in each occupation; and (3) leisure activity engagement, measured by the value of different activities regarding their frequency and periodicity. The total score of CRIq was then categorized into five levels: low (≤ 70), moderate low (70–84), moderate (85–114), moderate high (115–130), and high (≥ 130) (Nucci et al., 2012). CRIq had been translated into different languages and used in populations of Alzheimer’s disease, stroke, acquired brain injury (Bertoni et al., 2022), multiple sclerosis (Ozakbas et al., 2021), Parkinson’s disease (Guzzetti et al., 2019), and healthy adults (Maiovis et al., 2016). CRIq is a semi-structured interview questionnaire. If a patient is suspected to have cognitive deficits, a primary family member who knows the patient’s past and present living habits can be asked (Nucci et al., 2012). The Chinese version of the CRIq was translated and back-translated by our research team with three Ph.D.-prepared researchers in nursing and neuroscience who were fluent in both English and Chinese. There was no substantial change in the content of each item. Team members discussed each item and reached a consensus about the translation in order to ensure the accuracy of the translation and the culturally relevance of the Chinese version. The Cronbach’s α of CRIq in present study was 0.827 and the test-retest reliability was 0.995 (Li and Li, 2022).

#### Montreal Cognitive Assessment-Changsha version

Montreal Cognitive Assessment-Changsha Version (MoCA-CS) was used to assess cognitive function. The Montreal Cognitive Assessment (MoCA) has been widely used to screen for cognitive impairment in stroke or TIA patients (Nasreddine et al., 2005), and shown to have good sensitivity and specificity in detecting cognitive impairment (Schlegel et al., 2003; Tan et al., 2017). MoCA-CS was a Chinese version of MoCA (Tu et al., 2013)and it had been used to evaluate cognitive function in ischemic stroke patients (Ozdemir et al., 2001; Patel et al., 2002; Li et al., 2020). The Cronbach’s α for MOCA-CS is 0.884 (Tu et al., 2013). Same as the MoCA, the score of MoCA-CS ranged from 0 to 30, with higher score indicating better cognitive function. Patients who had no more than 6 years of education was added one additional point to the total score. A total score ≥ 27 indicates normal cognitive function, and < 27 indicates impaired cognitive function (Tu et al., 2013).

#### Stroke severity

The National Institutes of Health Stroke Scale (NIHSS) was used to evaluate baseline stroke severity at admission by neurologists. Higher scores of NIHSS indicate more severe levels of stroke (Adams et al., 1999; Kasner et al., 1999).

#### Covariates

Covariates included sociodemographic characteristics (i.e., age, gender, rural/urban residency, and marital status), classification of stroke [the Oxfordshire Community Stroke Project (OCSP) classification and the Trial of ORG 10172 in Acute Stroke Treatment (TOAST) classification] (Adams et al., 1993; Lindley et al., 1993), comorbidities (hypertension, diabetes, dyslipidemia, and atrial fibrillation) and stroke risk factors (smoking, drinking, cerebral vascular stenosis degree, and prior history of stroke). Smoking was operationalized as smoking more than 4 times per week for more than half a year (Yes/No) (GBD 2019 Stroke Collaborators, 2021). Alcohol consumption was measured by drinking alcohol at least once per week for more than half a year (Yes/No) (GBD 2019 Stroke Collaborators, 2021). The radiologists assessed the severity of intracranial/external artery stenosis on angiography. It was divided into three grades: mild (stenosis rate of < 30%), moderate (30∼69%), and severe (70∼99%) (Kernan et al., 2014; Vascular Surgery Group, Surgery Society of Chinese Medical Association, 2017). Covariates coding is provided in the Supplementary Table 1.

### Statistical analysis

Sociodemographic, clinical characteristics, stroke severity, cognitive reserve and cognitive function were described using mean and standard deviation or median and interquartile range for continuous variables and frequency and proportion for categorical variables. First, we conducted univariate linear regression analysis to explore the relationships among stroke severity (NIHSS), cognitive reserve, covariates and cognitive function. Then we performed Pearson/Spearman correlation analysis for those covariates which were significant (*p* < 0.1) in univariate linear regression. Only those covariates which were significant in univariate linear regression and had weak correlation with each other (*r* < 0.5, Supplementary Tables 3, 4) were allowed to enter into the multivariate linear regressions. The average years of education was 9 years (SD = 4.4). Because years of education was highly correlated with cognitive reserve (*r* = 0.832, *p* < 0.001), it was not included in the analysis. To assess the moderating effects of the cognitive reserve on the relationships between stroke severity and cognitive function, we performed a series of multivariate linear regression models as following (1) Model I was the base model adjusting for baseline NIHSS; (2) Model II: model I plus CRIq; (3) Model III: model II plus interaction between NIHSS and CRIq; (4) Model IV: model III plus covariates adjustment (i.e., age, marital status, OCSP classification, TOAST classification, cerebral vascular stenosis, diabetes, and atrial fibrillation). Variance inflation factor (VIF) values of all variables are provided to ensure that there is no collinearity among variables. Given previous reports of three-way interaction of age, years of education and lesion size on the stroke outcomes (Umarova et al., 2021), we had examined the three-way interaction of age, cognitive reserve and stroke severity on cognitive function. Sensitivity analysis was conducted by using categorical variables of cognitive reserve (CRIq ≤ 84, low; CRIq 85–114, moderate; and CRIq ≥ 115, high) and stroke severity (NIHSS < 5, mild stroke; NIHSS 5–15, moderate stroke; NIHSS > 15, severe stroke) (Kasner et al., 1999; Nucci et al., 2012). However, the three-way interaction of age, cognitive reserve and stroke severity was not significant. All analyses were conducted using the SPSS version 21.0. Statistical significance was set at *p* < 0.05.

## Results

**FIGURE 1:**
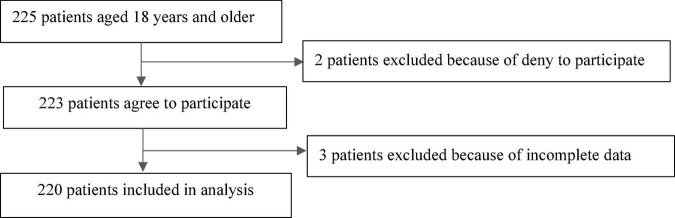
Flow chart of participants.

Figure 1 demonstrates the flowchart of patients. The characteristics of the participants are presented in Table 1. A total of 220 participants enrolled in this study. The average age of the participants was 67 years (SD: 9.9). Sixty five percent were male and 82.3% were married. The median score of NIHSS was 7 (IQR: 3–11). The mean score of CRIq was 99 (SD: 16.6). One hundred thirty-four participants (60.9%) had a moderate level of cognitive reserve. The median score of MoCA was 22 (IQR: 16–26) and 79.5% of the participants were cognitively impairment (MoCA < 27).

**TABLE 1:** Characteristics of patients with Acute Ischemic Stroke (*N* = 220).

	Variables	*M* (SD)/*N* (%)
Sociodemographic	Age(years)	67 (9.9)
characteristics	Gender(male)	143 (65.0%)
	Married	181 (82.3%)
	Years of education	9 (4.4)
	Rural residency	28 (12.7%)
Classification	OCSP classification	
of stroke	TACI	22 (10.0%)
	PACI	58 (26.4%)
	POCI	63 (28.6%)
	LACI	77 (35.0%)
	TOAST classification	
	LAA	70 (31.8%)
	CE	33 (15.0%)
	SAO	73 (33.2%)
	ODE	39 (17.7%)
	UDE	5 (2.3%)
Stroke risk	Smoking	94 (42.7%)
factors	Drinking	98 (44.5%)
	Previous history of stroke	48 (21.8%)
	Stenosis degree	
	Mild	107 (48.6%)
	Moderate	94 (42.7%)
	Severe	19 (8.6%)
Comorbidities	Hypertension	167 (75.9%)
	Diabetes	95 (43.2%)
	Dyslipidemia	45 (20.5%)
	Atrial fibrillation	32 (14.5%)
Severity of	NIHSS, median (IQR)	7 (3–11)
stroke	NIHSS < 5	77 (35.0%)
	NIHSS(5–15)	123 (55.9%)
	NIHSS > 15	20 (9.1%)
Cognitive	CRIq	99 (16.6)
reserve	CRI-education	103 (18.7)
	CRI-occupation	103 (14.1)
	CRI-leisure activity engagement	91 (9.3)
	Low (≤ 70)	7 (3.2%)
	Moderate low (70–84)	40 (18.2%)
	Moderate (85–114)	134 (60.9%)
	Moderate high (115–130)	34 (15.4%)
	High (≥ 130)	5 (2.3%)
Cognitive	MoCA-CS, median (IQR)	22 (16–26)
function	Cognitive impairment(MoCA < 27)	175 (79.5%)
	Normal cognition(MoCA ≥ 27)	45 (20.5%)

OCSP, Oxfordshire Community Stroke Project; TACI, total anterior circulation infarct; PACI, partial anterior circulation infarct; POCI, Posterior circulation infarct; LACI, Lacunar circulation infarcts; TOAST, Trial of ORG 10172 in Acute Stroke Treatment; LAA, Large-artery atherothrombotic; CE, Cardioembolic; SAO, Small-artery occlusion; ODE, Other determined etiology; UDE, Undetermined etiology; NIHSS, National Institutes of Health Stroke Scale; CRIq, Cognitive Reserve Index questionnaire; MoCA-CS, Montreal Cognitive Assessment-Changsha Version; *M(SD)* = Mean ± Standard Deviation; IQR, Interquartile.

Supplementary Table 2 presents the results from univariate linear regression analyses. Higher levels of cognitive reserve were associated with better performance in MoCA. Participants who were younger and married had higher scores in MoCA. More severe carotid artery stenosis, having comorbidities of diabetes and atrial fibrillation, and more severe stroke measured by NIHSS were significantly associated with lower scores in MoCA. Compared with participants who had Lacunar circulation infarcts (LACI) type of stroke, those with other Oxfordshire Community Stroke Project (OCSP) classifications exhibited significantly worse cognitive function. Participants with Small-artery occlusion (SAO) subtype had significant better cognitive performance on MoCA than those with Large-artery atherothrombotic (LAA) subtype.

**FIGURE 2:**
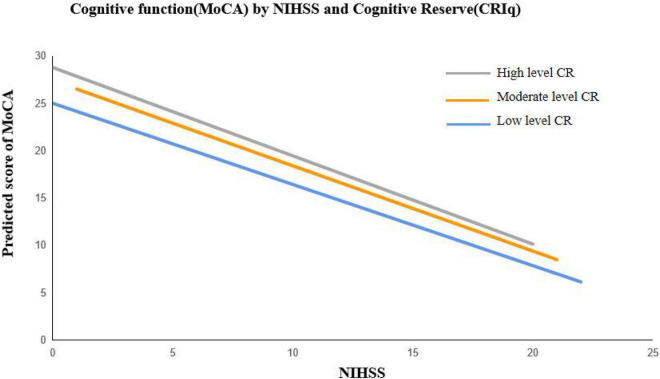
Cognitive function (MoCA) of AIS patients by stroke severity (NIHSS) and cognitive reserve. (CRIq) Cognitive reserve was stratified into low (CRIq ≤ 84), moderate (85 ≤ CRIq ≤ 114), and high (CRIq ≥ 115).

Table 2 presents the results from a series of linear regression models of stroke severity and cognitive reserve on cognitive function. After controlling for the covariates, higher level of stroke severity was associated with poorer cognitive function among AIS patients (β = −0.762, *p* < 0.001), higher level of cognitive reserve was related to better cognitive function (β = 0.074, *p* = 0.003). The interaction between NIHSS and CRIq was significant (β = −0.010, *p* = 0.045), which suggests that patients with higher level of cognitive reserve showed significant better cognitive function than those with lower level of cognitive reserve when stroke severity was mild (Figure 2). In addition, a control analysis including all variables at once in a multivariate regression confirmed the results, highlighting that the results are not bound the specific model selection procedure (Supplementary Table 5). Similar findings were observed in the sensitivity analyses (Supplementary Table 6 and Supplementary Figure 1). Patients with younger age (β = −0.116, *p* = 0.007) and those married had better cognitive function (β = 2.207, *p* = 0.040), whereas whose with diabetes had poorer cognitive function (β = −1.848, *p* = 0.031).

**TABLE 2:** Moderating effect of cognitive reserve on the relationship between stroke severity and cognitive function in patients with AIS.

				95% CI				
							
	Variables	B	*p*	Lower	Upper	VIF	Adjusted *R*^2^	*F*
Model I	NIHSS	–0.893	<0.001	–1.053	–0.734	1.000	0.356	122.047
Model II	NIHSS	–0.894	<0.001	–1.051	–0.737	1.000	0.375	66.585
	CRIq	0.067	0.007	0.019	0.116	1.000		
Model III	NIHSS	–0.902	<0.001	–1.058	–0.746	1.003	0.384	46.465
	CRIq	0.067	0.006	0.019	0.115	1.000		
	NIHSS × CRIq	–0.010	0.041	–0.020	0.000	1.003		
Model IV	NIHSS	–0.762	<0.001	–0.943	–0.581	1.454	0.427	11.214
	CRIq	0.074	0.003	0.026	0.121	1.049		
	NIHSS × CRIq	–0.010	0.045	–0.020	0.000	1.070		
	Age	–0.116	0.007	–0.199	–0.033	1.149		
	Married	2.207	0.040	0.104	4.309	1.095		
	OCSP classification							
	LACI (reference)							
	TACI	–0.922	0.577	–4.175	2.331	1.617		
	PACI	–1.170	0.356	–3.665	1.325	2.005		
	POCI	0.115	0.931	–2.484	2.714	2.344		
	TOAST classification							
	LAA (reference)							
	CE	–0.684	0.607	–3.299	1.932	1.481		
	SAO	–0.446	0.744	–3.135	2.244	2.723		
	ODE	0.396	0.742	–1.971	2.763	1.387		
	UDE	–5.476	0.114	–12.324	1.372	1.131		
	Cerebral vascular stenosis							
	Mild (reference)							
	Moderate	–0.417	0.665	–2.310	1.476	1.442		
	Severe	–2.775	0.112	–6.204	0.653	1.346		
	Diabetes	–1.848	0.031	–3.525	–0.171	1.172		
	Atrial fibrillation	–0.468	0.702	–2.877	1.940	1.224		

NIHSS, National Institutes of Health Stroke Scale; CRIq, Cognitive Reserve Index questionnaire; OCSP, Oxfordshire Community Stroke Project; TACI, Total anterior circulation infarct; PACI, Partial anterior circulation infarct; POCI, Posterior circulation infarct; LACI, Lacunar circulation infarcts; TOAST, Trial of ORG 10172 in Acute Stroke Treatment; LAA, Large-artery atherothrombotic; CE, Cardioembolic; SAO, Small-artery occlusion; ODE, Other determined etiology; UDE, Undetermined etiology; CI, Confidence Interval; VIF, Variance inflation factor; B, unstandardized coefficient; *p* < 0.05.

## Discussion

To the best of our knowledge, the present study was the first to explore the moderating effect of cognitive reserve on the relationship between stroke severity and cognitive function among Chinese patients with AIS. Stroke severity was negatively associated with cognitive function of AIS patients. Cognitive reserve plays a moderating effect on the relationship between stroke severity and cognitive function. After adjusting for covariates, patients with higher level of cognitive reserve had better cognitive function, especially among patients with mild stroke.

There was little relevant research about cognitive reserve conducted in China. The cognitive reserve theory has been widely tested in western population, but not in Chinese population (Du and Qiu, 2019). The findings of our study were consistent with results from previous studies that cognitive reserve could buffer the cognitive impairment after stroke (Shin et al., 2020; Umarova et al., 2021). Unlike prior research that used education and/or occupation as the proxy for cognitive research, our study assessed cognitive reserve using a comprehensive and validated instrument that consists of education, occupation and leisure activities engagement (Gil-Pagés et al., 2019; Kartschmit et al., 2019), which further confirm the buffering effect of cognitive reserve on cognitive impairment in AIS patients. In addition, we found that older age, higher score of stroke severity and diabetes were associated with poor post-stroke cognition, which were in line with previous findings (Pendlebury and Rothwell, 2009; Ravona-Springer et al., 2010; Ramsey et al., 2017).

The cognitive reserve theory may provide explanations on the moderating effects of cognitive reserve on cognitive function. Cognitive reserve has an additive effect (Stern, 2012)—patients with higher cognitive reserve generally have better premorbid cognitive performance and could tolerate more severe brain pathological damage, and therefore present a better cognitive function after stroke. On the other hand, individuals with higher levels of cognitive reserve may have more effective cognitive processing strategies and exhibit increased neuroplasticity (Barulli and Stern, 2013). As a result, when individuals with high cognitive reserve suffer from a stroke, they are able to leverage alternative neural networks that are unimpaired to maintain a relative high level of cognitive functioning (Steffener et al., 2011). However, among patients with severe stroke (NIHSS > 15), AIS patients with higher levels of cognitive reserve had worse cognitive function. It may be explained that the moderating effect of cognitive reserve was weakened in patients with severe brain pathological damage (Stern, 2009).

The incidence and disease burden of stroke are increasing in low and middle-income countries due to substantial population aging (Mijajlović et al., 2017). Cognitive reserve shows great potential in reducing the burden of stroke-induced cognitive impairment and/or vascular dementia. Prior research has suggested that cognitive reserve is dynamic and can be accumulated across the lifespan (Stern, 2012)through engaging in educational, occupational, and leisure activities (Wang H. X. et al., 2017). Participating in multiple social activities at different life stages may have different effects on the development and accumulation cognitive reserve. Prior research has suggested that cognitive activities in childhood (including educational, reading and other intelligent recreations) have a positive impact on cognition and cognitive reserve in adulthood (Richards and Sacker, 2003), occupational attainment in midlife protect the structural brain integrity and health and cognitive ability in older ages (Chan et al., 2018), and physical exercise and social activities in later life delay the onset of dementia (Fratiglioni et al., 2004). Even after the onset of cognitive impairment, enhancement of cognitive reserve still has the effect of delaying further cognitive decline (Dekhtyar et al., 2015; Wang H. X. et al., 2017), due to the additive effect of cognitive reserve. For AIS patients, engagement in evidence-based physical and social activities after stroke is critical to increase cognitive reserve and to reduce cognitive impairment or remain cognitive function.

This study also had several limitations: (1) Due to the difficulty in obtaining neuropathological data, we adopted the NIHSS score as severity of stroke pathological damage. However, NIHSS is an indicator of pathological damage for stroke patients and it has been widely used in stroke studies (Adams et al., 1999; Umarova, 2017). (2) We did not collect information on the infarct locations and psychiatric factors (such as depression, anxiety, sleep disorders, and fatigue, etc.) in this study although they may be associated with cognitive function after stroke (Ismail et al., 2018; Weaver et al., 2021). We will address these issues in future studies. (3) This study was a cross-sectional study using the convenience sampling method, and the sample may not be representative enough for the whole Chinese AIS population. Future studies with larger sample sizes are needed to further assess the role of cognitive reserve in severe stroke patients. (4) The cross-sectional study design prohibited us from examining the effects of cognitive reserve on the trajectory of cognitive decline over time. Longitudinal studies are needed to investigate the protective effect of cognitive reserve on cognitive function after stroke in future.

## Conclusion

Our findings suggest that cognitive reserve plays a moderating role in the relationship between brain pathological damage caused by stroke and cognitive function in AIS patients. Higher level of cognitive reserve is associated with better cognitive function especially in mild stroke patients. Interventions that are designed to improve social activities would be beneficial for cognitive reserve, and in turn, may have the potential to delay post-stroke cognitive decline.

## Data availability statement

The original contributions presented in this study are included in the article/Supplementary material, further inquiries can be directed to the corresponding authors.

## Ethics statement

The studies involving human participants were reviewed and approved by the Institutional Review Board (IRB) of Naval Medical University (NMUMREC-2021-001). The patients/participants provided their written informed consent to participate in this study.

## Author contributions

FL suggested the analytical strategy, performed data analysis, and wrote the manuscript. XK and HZ collected the data and interpreted the data. HX interpreted the study findings and drafted the manuscript. JL contributed to concept formation and design of the study protocol, interpretation of data, and preparation of the manuscript. BW provided input on the conceptualization of the study, interpreted the study findings, and reviewed the manuscript. YC provided input on the study design and reviewed the manuscript. All authors contributed to the article and approved the submitted version.
