# Supplementary material for: The moderating effect of cognitive reserve on cognitive function in patients with Acute Ischemic Stroke
Source: Front Aging Neurosci. 2022 Nov 16;14:1011510. doi: 10.3389/fnagi.2022.1011510 (PMC9710856; doi:10.3389/fnagi.2022.1011510)
Supplement: Supplementary file 1 [file Data_Sheet_1.pdf]

Appendix

Supplementary Materials:

Table 1 The list of covariates coding

Covariates	Categorical variables	Coding
Sociodemographic characteristics	Gender	Male = 1, female = 0
	Rural/Urban Residency	Urban = 1, Rural = 0
	Marital status	Married=1, others=0
Classification of stroke	Oxfordshire Community Stroke Project (OCSP) Classifification	
	Lacunar circulation infarcts (LACI)	LACI=Reference
	Total anterior circulation infarct (TACI)	
	Partial anterior circulation infarct (PACI)	
	Posterior circulation infarct (POCI)	
	Trial of ORG 10172 in Acute Stroke Treatment (TOAST) Classification	
	Large-artery atherothrombotic (LAA)	LAA=Reference
	Cardioembolic (CE)	
	Small-artery occlusion (SAO)	
	Other determined etiology (ODE)	
	Undetermined etiology (UDE)	
Comorbidities	Hypertension	Hypertension=1, no=0
	Diabetes	Diabetes=1, no=0
	Dyslipidemia	Dyslipidemia=1, no=0
	Atrial fibrillation	Atrial fibrillation=1, no=0
Stroke risk factors	Cerebral vascular stenosis	
	Mild	Mild=Reference
	Moderate	
	Severe	
	Smoking	Smoking=1, no=0
	Drinking	Drinking=1, no=0
	Prior history of stroke	Prior history of stroke=1, no=0

Table 2 Univariate linear regression for cognitive function of AIS patients

Variables	B	95% CI		*p*
		Lower	Upper	
Gender(Male)	0.674	-1.454	2.803	0.533
Age	-0.141	-0.242	-0.040	0.006
Residency (Urban)	2.281	-0.753	5.314	0.140
Marital status（Married）	4.020	1.414	6.626	0.003
OCSP classification				
LACI（Reference）				
TACI	-3.690	-7.265	-0.115	0.043
PACI	-3.476	-6.066	-0.885	0.009
POCI	-2.555	-5.06	-0.050	0.046
TOAST classification				
LAA（Reference）				
CE	0.339	-2.770	3.447	0.830
SAO	3.366	0.903	5.829	0.008
ODE	0.504	-2.438	3.446	0.736
UDE	-6.086	-12.901	0.729	0.080
Cerebral vascular stenosis				
Mild（Reference）				
Moderate	-3.812	-5.847	-1.776	＜0.001
Severe	-7.673	-11.489	-3.856	＜0.001
Hypertension	-1.595	-3.961	0.772	0.185
Diabetes	-3.621	-5.614	-1.627	＜0.001
Dyslipidemia	-1.458	-3.970	1.053	0.254
Atrial fibrillation	-4.189	-7.016	-1.362	0.004
Smoking	-0.079	-2.132	1.975	0.940
Drinking	0.094	-2.082	2.007	0.928
Previous history of stroke	-1.141	-3.596	1.314	0.361
NIHSS	-0.893	-1.053	-0.734	＜0.001
CRIq	0.067	0.006	0.128	0.031

Note:NIHSS=National Institutes of Health Stroke Scale; OCSP=Oxfordshire Community Stroke Project; TACI=Total anterior circulation infarct; PACI=Partial anterior circulation infarct; POCI=Posterior circulation infarct; LACI=Lacunar circulation infarcts; TOAST=Trial of ORG 10172 in Acute Stroke Treatment; LAA=Large-artery atherothrombotic; CE=Cardioembolic; SAO=Small-artery occlusion; ODE= Other determined etiology; UDE=Undetermined etiology; CRIq=Cognitive Reserve Index questionnaire; CI=Confidence Interval; B=Unstandardized Coefficient.

Table 3 Pearson correlation between Cognitive Reserve, NIHSS, and Age

Variables	NIHSS	Age
CRIq	0.001	-0.007
NIHSS	—	0.059
Age		—

Note: CRIq=Cognitive Reserve Index questionnaire; NIHSS=National Institutes of Health Stroke Scale; *Correlation is significant at $p<0.05$ level. ** Correlation is significant at $p < 0.01$ level.

Table 4 Spearman correlation between Marital status, OCSP classification, TOAST classification, Cerebral vascular stenosis, Diabetes, and Atrial fibrillation

Variables	OCSP classification	TOAST classification	Cerebral vascular stenosis	Diabetes	Atrial fibrillation
Marital status	0.047	0.129	-0.008	-0.076	-0.045
OCSP classification	-	0.212**	-0.209**	-0.107	-0.038
TOAST classification		-	-0.180*	0.045	-0.182**
Cerebral vascular stenosis			-	0.078	0.162*
Diabetes				-	0.161*
Atrial fibrillation					-

Note: OCSP=Oxfordshire Community Stroke Project; TOAST=Trial of ORG 10172 in Acute Stroke Treatment; *Correlation is significant at $p< 0.05$ level. ** Correlation is significant at $p <0.01$ level.

Table 5 Multivariate regression with all variables included

Variables	B	*P*	95% CI		VIF
			lower	upper	
NIHSS	-0.803	＜0.001	-0.993	-0.613	1.583
CRIq	0.057	0.037	0.003	0.110	1.300
NIHSS * CRIq	-0.011	0.032	-0.021	-0.001	1.149
Gender(Male)	1.847	0.111	-0.425	4.119	1.972
Age	-0.135	0.003	-0.223	-0.046	1.291
Residency (Urban)	1.343	0.314	-1.284	3.971	1.288
Married	1.682	0.132	-0.512	3.876	1.179
OCSP classification					
LACI（Reference）					
TACI	-0.951	0.569	-4.242	2.340	1.637
PACI	-1.348	0.305	-3.931	1.235	2.127
POCI	-0.247	0.856	-2.933	2.438	2.475
TOAST classification					
LAA（Reference）					
CE	-1.362	0.332	-4.126	1.402	1.636
SAO	-1.029	0.470	-3.833	1.775	2.927
ODE	0.226	0.855	-2.200	2.652	1.442
UDE	-6.169	0.033	-11.82	-0.518	1.191
Cerebral vascular stenosis					
Mild（Reference）					
Moderate	-0.557	0.570	-2.486	1.372	1.482
Severe	-2.302	0.202	-5.851	1.247	1.427
Hypertension	0.138	0.889	-1.822	2.099	1.181
Diabetes	-1.845	0.038	-3.588	-0.102	1.252
Dyslipidemia	1.048	0.341	-1.117	3.213	1.281
Atrial fibrillation	-0.864	0.517	-3.489	1.761	1.438
Smoking	-1.567	0.162	-3.768	0.634	1.991
Drinking	0.203	0.840	-1.774	2.180	1.622
Previous history of stroke	0.292	0.784	-1.812	2.397	1.269

Note: NIHSS=National Institutes of Health Stroke Scale; CRIq=Cognitive Reserve Index questionnaire; OCSP=Oxfordshire Community Stroke Project; TACI=Total anterior circulation infarct; PACI=Partial anterior circulation infarct; POCI=Posterior circulation infarct; LACI=Lacunar circulation infarcts; TOAST=Trial of ORG 10172 in Acute Stroke Treatment; LAA=Large-artery atherothrombotic; CE=Cardioembolic; SAO=Small-artery occlusion; ODE= Other determined etiology; UDE=Undetermined etiology; CI=Confidence Interval; B=Unstandardized Coefficient; VIF=Variance Inflation Factor.

Table 6 Sensitivity analysis of cognitive function (MoCA) by NIHSS and Cognitive Reserve (CRIq)

Variables	B	95%CI Lower	95%CI upper	*p*
NIHSS＜5（Reference）				
NIHSS 5-15	-3.938	-5.683	-2.192	＜0.001
NIHSS＞15	-15.560	-18.536	-12.584	＜0.001
CRIq≤84（Reference）				
CRIq 85-114	2.001	0.019	3.983	0.048
CRIq ≥115	3.957	1.396	6.517	0.003
Age	-0.141	-0.223	-0.060	0.001
Married	1.845	-0.244	3.933	0.083
Diabetes	-2.401	-4.069	-0.734	0.005

Note: NIHSS=National Institutes of Health Stroke Scale; CRIq=Cognitive Reserve Index questionnaire; CI=Confidence Interval; B=Unstandardized Coefficient.

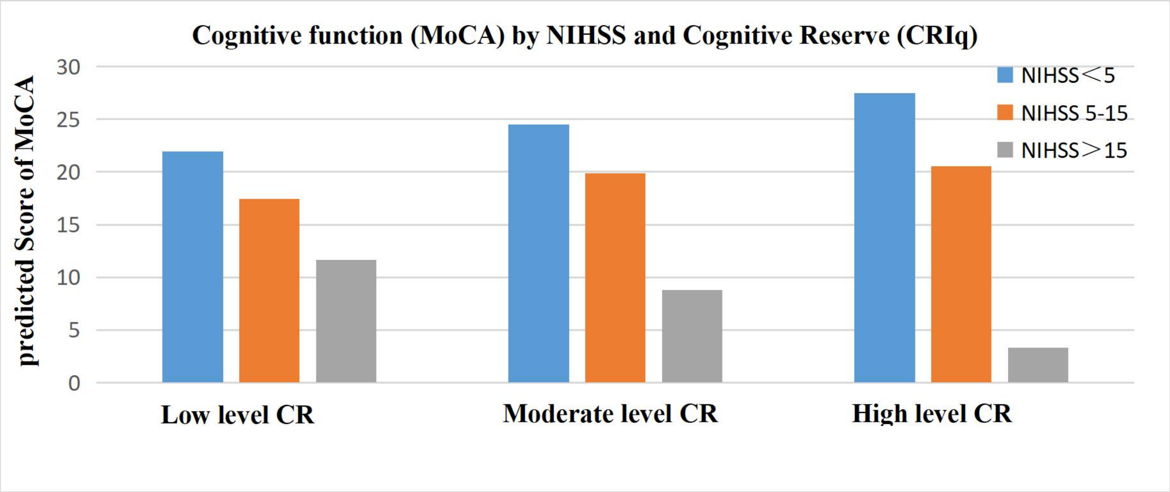

Figure 1. Visual representations showing the moderating effect of cognitive reserve (CRIq) on the relationship between stroke severity (NIHSS) and cognitive function (MoCA). Stroke severity was stratified into low (NIHSS<5), moderate (5≤NIHSS≤15) and high (NIHSS>15). Cognitive reserve was stratified into low (CRIq≤84), moderate (85≤CRIq≤114) and high (CRIq≥115).
